# *Spirulina platensis* Alleviated the Hemotoxicity, Oxidative Damage and Histopathological Alterations of Hydroxychloroquine in Catfish (*Clarias gariepinus*)

**DOI:** 10.3389/fphys.2021.683669

**Published:** 2021-07-06

**Authors:** Alaa El-Din H. Sayed, Mohamed Hamed, Hamdy A. M. Soliman

**Affiliations:** ^1^Department of Zoology, Faculty of Science, Assiut University, Assiut, Egypt; ^2^Department of Zoology, Faculty of Science, Al Azhar University (Assiut Branch), Assiut, Egypt; ^3^Department of Zoology, Faculty of Science, Sohag University, Sohag, Egypt

**Keywords:** *Spirulina*, hydroxychloroquine, *Clarias gariepinus*, AST, TAC

## Abstract

The current study aims at evaluating the toxicity of hydroxychloroquine (HCQ) as a pharmaceutical residue in catfish (*Clarias gariepinus*) and the protective role of *Spirulina platensis* (SP). Four groups were used in this study: (1) a control group, (2) a group exposed to 3.16 mg/l of HCQ, (3) a group exposed to 3.16 mg/l of HCQ + 10 mg/l of SP, and (4) a group exposed to 3.16 mg/l of HCQ + 20 mg/l of SP for 15 days of exposure. The HCQ-treated group showed a significant decline in the hematological indices and glucose, total protein, and antioxidant levels in relation to the control group, whereas the HCQ-treated group showed a significant increase in the levels of creatinine, uric acid, aspartate aminotransferase (AST), and alanine aminotransferase (ALT) as well as the percentage of poikilocytosis and nuclear abnormalities of RBCs in relation to the control group. The histopathological evaluation of the liver indicated dilation of the central vein, vacuolization, degeneration of hepatocytes and pyknotic nuclei, as well as reduction of glomeruli, dilation of Bowman’s space, and degeneration of renal tubules in the kidney of the HCQ-treated group. *Spirulina platensis* (SP) rendered the hematological and biochemical indexes as well as antioxidant levels and the histological architecture to normal status in a dose-dependent manner. Accordingly, the current study recommends the use of SP to remedy the toxic effects of HCQ.

## Highlights

- Hydroxychloroquine (HCQ) induced changes in biochemical and antioxidant parameters.
- Hydroxychloroquine (HCQ) prompted abnormalities in erythron profile of catfish
- Hydroxychloroquine (HCQ) induces damages in the histopathological structure of catfish
- *Spirulina* supplementation protects against Hydroxychloroquine (HCQ) deleterious effects.

## Introduction

The increasing global use of pharmaceutical and personal-care products is leading to the increasing contamination of surface water and groundwater, which raises a significant ecotoxicological concern (Schwarzenbach et al., 2006; Hughes et al., 2013; Margot et al., 2015). Conventional wastewater treatment plants (WWTPs) are designed to remove solids, suspended solids, and easily (bio-)degradable organic material. Consequently, many of these pharmaceutical products are only partially removed in current WWTPs (Lindroos et al., 2019). This problem is especially significant in developing countries, where untreated effluents from the hospitals and homes result in the release of high quantities into the environment (Larsson et al., 2007; Carlsson et al., 2009; Rana et al., 2017).

Hydroxychloroquine (HCQ) is used in the treatment protocols of malaria and various inflammatory diseases like rheumatoid arthritis and systemic lupus erythematosus (Borba et al., 2004), as well as management of a number of viral diseases, such as influenza A/H5N1, SARS-CoV, and HIV (Takano et al., 2013). Recently, national and international medical organizations over the world allowed the treatment of Coronavirus (COVID-19) in certain hospitalized patients with chloroquine and HCQ. This certainly results in the discharge of large quantities of wastewaters contaminated with HCQ into the environment (Bensalah et al., 2020). The behavior and fate of pharmaceutical HCQ in the aquatic environment are significantly unexplored (Kumar et al., 2021).

HCQ has a serious chronic threat to the aquatic environment as the drug belongs to a group of quinolone derivatives that are recalcitrant, toxic, persistent, teratogenic, and carcinogenic for the aquatic organisms (Ramesh et al., 2018). Although the mechanism of chloroquine or HCQ toxic effects has not been precisely identified yet, some researchers are pointing at the formation of reactive oxygen species (El Shishtawy et al., 2013).

*Spirulina* is a freshwater microscopic single-cell microalga (Sharoud, 2015) that contains antioxidant enzymes with the ability to downregulate free radical generation (Seshadri et al., 1991). Furthermore, *Spirulina platensis* (SP) has been verified to protect against the toxicity of some known drugs, such as paracetamol (Sharoud, 2015), D-galactosamine, acetaminophen (Sabina et al., 2009; Lu et al., 2010; Zeenat et al., 2017), methotrexate (Khafaga and El-Sayed, 2018), aspirin (Mahmoud and Abd El-Ghffar, 2019), isoniazid, and rifampicin (Martin and Sabina, 2016). However, little is known about the potential protective mechanisms of *Spirulina* against HCQ toxicity in fish and other animals.

Catfish (*Clarias gariepinus*) is used in toxicology and bioremediation research due to its high growth rate, and hardiness, including its high resistance to handling and stress (Sayed and Soliman, 2018). The current study aims to evaluate the toxicity of HCQ as pharmaceutical residue in catfish (*Clarias gariepinus*) and the protective role of SP.

## Materials and Methods

### Chemicals

HCQ [Plaquenil, white, round, film-coated 200-mg tablets, Sanofi^®^] was obtained from a drug store. A stock solution of HCQ was prepared (3,000 mg/l deionized water).

*SP* tablets were bought from Japan Algae Co., Ltd., *Spirulina* contains proteins, sugariness, fatty acids, and vitamins, as well as plenty of minerals, such as calcium, magnesium, iron, and zinc. *Spirulina* tablets dissolved in water, and their bioactive ingredients became available in the water column for fish to absorb *via* the gastrointestinal canal.

### Fish Exposure

The fish *Clarias gariepinus* (weight of 478.85 ± 3.03 g and a mean length of 32 ± 0.3 cm, *n* = 96, male) were obtained from a fish farm at Assiut University and transported to the Fish Biology and Pollution Laboratory, Assiut University. They were healthy and parasite-free according to AFS-FHS (2004). The fish were kept in ≈200-l glass tanks (92 cm × 46 cm × 46 cm) containing 100 l dechlorinated tap water and air pumps under laboratory circumstances for 4 weeks for acclimatization. The physicochemical properties of the test water were recorded as follows: conductivity 260.8 μM cm^–1^, pH 7.56, dissolved oxygen 6.9 mg l^–1^, temperature 29.5°C, and photoperiod 12:12 light:dark. Four groups (24 fish/group) were assigned in three replicates for each treatment group (eight fish/glass aquarium according to Cash et al., 2016) during the experimental period. The first group was a control group; the second group were exposed to 3.16 mg/l of HCQ according to Ramesh et al. (2018)—this concentration is lower than LC_50_ > 100 mg/l according to SANOFI (2020); the third group was exposed to 3.16 mg/l of HCQ + 10 mg/l of SP; and the fourth group was exposed to 3.16 mg/l of HCQ + 20 mg/l of SP for 15 days. After the exposure, six fish per replicate from each group were randomly selected and anesthetized with ice (Hamed et al., 2019a). Blood samples (1.5 ml) were collected from the caudal vein, one part for the erythron profile and hematological indices, and the other part centrifuged under cooling for biochemical and antioxidant biomarkers. Liver and kidney tissues were used for histopathological studies. The experimental setup, guidelines, and fish handling were approved by the Research and Ethical Committee of the Faculty of Science, Assiut University, Assiut, Egypt.

### Hemato-Biochemical Parameters

The hematological parameters, i.e., white blood cell count (WBC), red blood cell count (RBC), thrombocytes, hemoglobin (Hb), hematocrit (HCT), mean corpuscular hemoglobin (MCH), mean corpuscular volume (MCV), and mean corpuscular hemoglobin concentration (MCHC), were performed according to Fazio et al. (2014). Likewise, biochemical parameters, i.e., uric acid, creatinine, glucose, ALT, AST, and total protein, were assessed as described by Hamed et al. (2019a) using a spectrophotometer (Jasco V-530, Ottawa, Canada).

### Measurement of Antioxidant Biomarkers

Superoxide dismutase (SOD) was measured based on its ability to inhibit the phenazine methosulfate-mediated reduction of nitroblue tetrazolium dye to form a red product (Nishikimi et al., 1972). Catalase (CAT) was determined based on the fact that 3,5-dichloro-2-hydroxybenzene sulfonic acid could rapidly terminate the degradation reaction of hydrogen peroxide catalyzed by CAT and react with the residual hydrogen peroxide to generate a yellow product (Aebi, 1984). The TAC assay measured the capacity of the biological fluids to inhibit the production of thiobarbituric acid reactive substances (TBARS) from sodium benzoate under the influence of the free oxygen radicals derived from Fenton’s reaction. A solution of 1 mmol/l uric acid was used as standard (Koracevic et al., 2001).

### Erythron Profile (Poikilocytosis and Nuclear Abnormalities of RBCs)

Blood smears were prepared, stained with hematoxylin–eosin, selected, coded, randomized, and scored blindly for erythrocyte alterations and nuclear abnormalities following the criteria of Schmid (1975) and Al-Sabti and Metcalfe (1995).

### Histopathological Studies

The liver and kidney samples were taken and then fixed in 10% neutral buffered formalin. Fixed samples were processed routinely using a paraffin embedding technique, then sectioned at 5 μm in thickness and stained with Harris’ hematoxylin and eosin (H&E). Sections were examined using an Olympus microscope model BX50F4 (Olympus Optical Co., Ltd., Tokyo, Japan).

### Statistical Analysis

Data were analyzed using the SPSS package (SPSS, 1998), and 0.05 was considered the point of significance. Data were then tested for normality (Shapiro–Wilk test). Then, the homogeneity of variances was tested (Levene’s test) following the one-way analysis of variance (ANOVA). Fisher’s LSD *post hoc* test was used in case of variance equality to compare the treated groups in relation to the control group. Dunnett’s *post hoc* test was used in case of variance inequality to compare the treated groups in relation to the control group.

## Results

### Hematological Parameters

The hematological indices showed a significant decrease (*P* < 0.05) after exposure to 3.16 mg/l of HCQ for 15 days in comparison with the control group, whereas MCV, MCHC, and MCH showed a non-significant increase (*P* < 0.05) after exposure to 3.16 mg/l of HCQ for 15 days. Co-treatment with *Spirulina* significantly improved the hematological indices which were decreased by HCQ in a dose-dependent manner (Table 1).

**TABLE 1:** Hematological, biochemical parameters, percentage of poikilocytosis, and nuclear abnormalities of the African catfish (*Clarias gariepinus*) after hydroxychloroquine (HCQ) exposure for 15 days and treatment with SP (in 10 and 20 mg l^–1^ water).

Parameters Groups	Control	Hydroxychloroquine	Hydroxychloroquine + 10 mg/l *Spirulina*	Hydroxychloroquine + 20 mg/l *Spirulina*
**Hematological parameters**				
(RBCs) (million/mm^3^)	3.2 ± 0.0^a^	2.8 ± 0.0^b^	2.9 ± 0.1^c^	3.1 ± 0.1^ac^
Hemoglobin (Hb) (g/dl)	9.6 ± 0.1^a^	8.5 ± 0.1^b^	8.9 ± 0.1^c^	9.2 ± 0.1^d^
Ht (PCV) (%)	35.4 ± 0.1^a^	32.8 ± 0.2^b^	34.6 ± 0.8^ab^	35.9 ± 0.4^a^
MCV (μm^3^)	111 ± 1.6^a^	117 ± 1.8^a^	117 ± 3.9^a^	118 ± 2.9^a^
MCH (pg)	30.1 ± 0.4^a^	30.4 ± 0.5^a^	30.1 ± 0.3^a^	30.2 ± 0.2^a^
MCHC (%)	26.9 ± 0.2^a^	25.9 ± 0.1^a^	25.7 ± 0.8^a^	25.6 ± 0.6^a^
Thrombocytes (thou./mm^3^)	214 ± 0.7^a^	202 ± 2.1^b^	208 ± 0.6^b^	210 ± 0.3^ab^
(WBCs) (thou./mm^3^)	11.3 ± 0.0^a^	10.5 ± 0.1^b^	10.9 ± 0.1^c^	10.9 ± 0.1^c^
**Biochemical parameters**				
Creatinine (mg/dl)	0.35 ± 0.01^a^	0.39 ± 0.0^b^	0.36 ± 0.0^a^	0.34 ± 0.0^a^
Uric acid (mg/dl)	2.3 ± 0.0^a^	2.6 ± 0.1^b^	2.4 ± 0.0^c^	2.3 ± 0.0^a^
AST (μ/l)	34.1 ± 0.9^a^	37.7 ± 0.4^b^	35.6 ± 0.4^a^	34.9 ± 0.2^a^
ALT (μ/l)	17.3 ± 0.3^a^	19.7 ± 0.2^b^	18.6 ± 0.1^a^	17.3 ± 0.4^a^
Glucose (mg/dl)	88.1 ± 0.3^a^	73.3 ± 2.6^b^	77.9 ± 1.1^c^	82.6 ± 1.3^d^
Total protein (mg/dl)	4.2 ± 0.2^a^	3.4 ± 0.1^b^	4 ± 0.1^a^	3.6 ± 0.1^b^
**Poikilocytosis %**				
Hemolyzed cell	2.3 ± 0.3^a^	8.3 ± 0.3^b^	4.7 ± 0.7^c^	4.7 ± 0.3^c^
Sickle cell	0.7 ± 0.3^a^	4.3 ± 0.3^b^	2.7 ± 0.3^c^	1.7 ± 0.3^ac^
Irregular shapes	0.3 ± 0.3^a^	12.3 ± 0.3^b^	4.7 ± 0.3^c^	3.3 ± 0.9^c^
Schistocyte	0.3 ± 0.3^a^	6.7 ± 0.7^b^	4 ± 0.6^c^	4 ± 0.6^c^
Acanthocyte	1.3 ± 0.3^a^	6.7 ± 0.9^b^	7 ± 0.6^b^	3 ± 0.6^c^
Tear drop	1 ± 0^a^	5.3 ± 0.9^b^	5 ± 0.6^bc^	3.3 ± 0.3^c^
Heinz bodies	1 ± 0.6^a^	1.7 ± 0.3^a^	2.7 ± 0.7^a^	1.3 ± 0.3^a^
Elliptocyte	1.3 ± 0.3^a^	8 ± 0.6^b^	6.7 ± 0.3^b^	3.3 ± 0.7^c^
Heart shape	0.3 ± 0.3^a^	1.3 ± 0.3^a^	0.7 ± 0.3^a^	0.3 ± 0.3^a^
Eccentric nucleus	2 ± 0.6^a^	18.7 ± 0.3^b^	11.3 ± 0.3^c^	6.3 ± 0.7^d^
Crenated cell	2.3 ± 0.3^a^	9.7 ± 0.9^b^	7.3 ± 0.3^c^	3.7 ± 0.3^a^
Kidney shape	0.3 ± 0.3^a^	1.7 ± 0.3^b^	1.3 ± 0.3^ab^	0.7 ± 0.3^ab^
**Nuclear abnormalities**				
Micronuclei	0.7 ± 0.3^a^	9.3 ± 0.9^b^	6.3 ± 0.9^c^	5.3 ± 0.7^c^
Binucleated	0.7 ± 0.3^a^	8.3 ± 0.7^b^	5.7 ± 0.9^c^	4.3 ± 0.3^c^
Blebbed nuclei	0 ± 0^a^	2.3 ± 0.3^b^	1.7 ± 0.3^bc^	1.3 ± 0.3^c^
Notched nuclei	0.3 ± 0.3^a^	4.7 ± 0.7^b^	2.3 ± 0.3^c^	1.7 ± 0.3^ac^
Lobed nuclei	0. ± 0^a^	2.3 ± 0.7^b^	1.3 ± 0.3^b^	1 ± 0^a^

**Data are represented as means ± SE. Values with a different superscript letter in the same row for each parameter are significantly different (P < 0.05). n = 6. RBCs, red blood cells; Hb, hemoglobin concentration; HCT, hematocrit; MCV, mean cell volume; MCH, mean cell hemoglobin; MCHC, mean cell hemoglobin concentration; PL, platelets; WBCs, white blood cells; AST, aspartate aminotransferase; ALT, alanine aminotransferase; poikilocytosis, alteration of erythrocyte shape.**

### Biochemical Parameters

Kidney functions, i.e., uric acid and creatinine, as well as liver functions, i.e., AST and ALT, showed a significant increase (*P* < 0.05) after exposure to 3.16 mg/l of HCQ for 15 days compared to the control group, whereas other biochemical parameters, like total protein and glucose, showed a significant decrease (*P* < 0.05) after exposure to 3.16 mg/l of HCQ for 15 days compared to the control group (Table 1). Co-treatment with *Spirulina* significantly rendered these biochemical parameters to their normal levels induced by HCQ in a dose-dependent manner (Table 1).

### Antioxidant Enzymes

**FIGURE 1:**
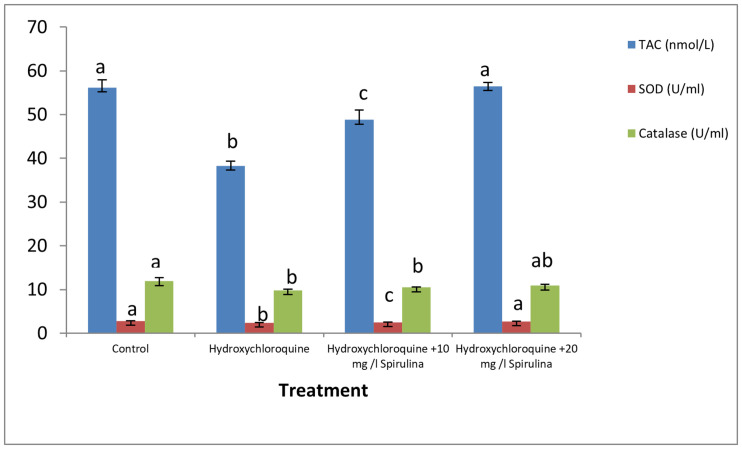
Effect of hydroxychloroquine (HCQ) exposure for 15 days on antioxidant parameters of the African catfish (*Clarias gariepinus*) and treatment with SP (in 10 and 20 mg l^– 1^ water). Bars with different superscript letters for each parameters are significantly different (*P* < 0.05).

The levels of antioxidant biomarkers, i.e., TAC, CAT, and SOD, were significantly depleted in the catfish treated with HCQ. Co-treatment with *Spirulina* significantly increased the levels of antioxidants in HCQ-treated groups in a dose-dependent manner (Figure 1).

### Erythron Profile

**FIGURE 2:**
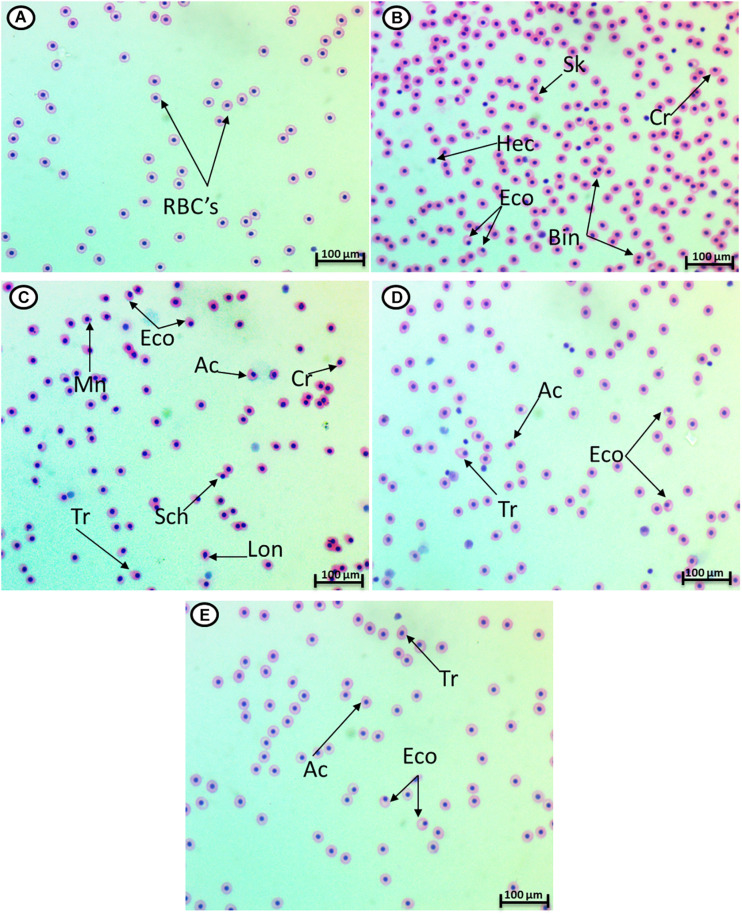
Hematoxylin–eosin-stained blood smears from catfish showing I: the normal erythrocytes **(A)**, the deformed ones after exposure to hydroxychloroquine for 15 days **(B,C)**, the deformed ones after exposure to hydroxychloroquine + 10 mg/l *Spirulina* for 15 days **(D)**, and the deformed ones after exposure to hydroxychloroquine + 20 mg/l *Spirulina* for 15 days **(E)**. Tr, tear-drop cell; Cr, crenated cell; Ac, acanthocyte; Eco, eccentric nucleus; Hec, hemolyzed cells; Mn, micronucleus; Bin, bionuclei; Lon, lobed nucleus; Shc, schistocytes; Sk, sickle cell (magnification: 1,000×).

HCQ (3.16 mg/l) caused a significant increase in the percentage of poikilocytosis and nuclear abnormalities of RBCs in relation to the control group (Table 1). The blood smears of control fish showed erythrocytes, which are ellipsoidal in shape with a centrally located ellipsoidal nucleus (Figure 2A). The blood smear of HCQ-treated catfish (*C. gariepinus*) showed poikilocytosis of erythrocytes. The major alterations of RBCs are teardrop-like cells (Tr), whose shape looks like a tear with pointed apices; sickle cells (Sk), which are elongated, crescent-shaped RBCs; eccentric nuclei, which are nuclei deviating or departing from the center of the cell; acanthocytes (Ac), where the red blood cells develop an irregular cell surface with numerous projections; crenated cells (Cr); with fewer projections from the surface; schistocytes (fragmented RBCs); and hemolyzed cells (Figures 2B,C). Likewise, there are still some alterations of RBCs in the fish exposed to HCQ and co-treated with *Spirulina* (10 and 20 mg/l), such as tear drop-like cells, acanthocytes, and eccentric nuclei (Figures 2D,E).

The major alterations of nuclei of RBCs in the fish treated with HCQ are shown by micronuclei (Mn), binuclei (Bin), and lobed nucleus (Lon) and having a large invagination of the nuclear envelope that has no clear shape or definition (Figures 2B,C).

### Hepato-Nephropathological Alterations

**FIGURE 3:**
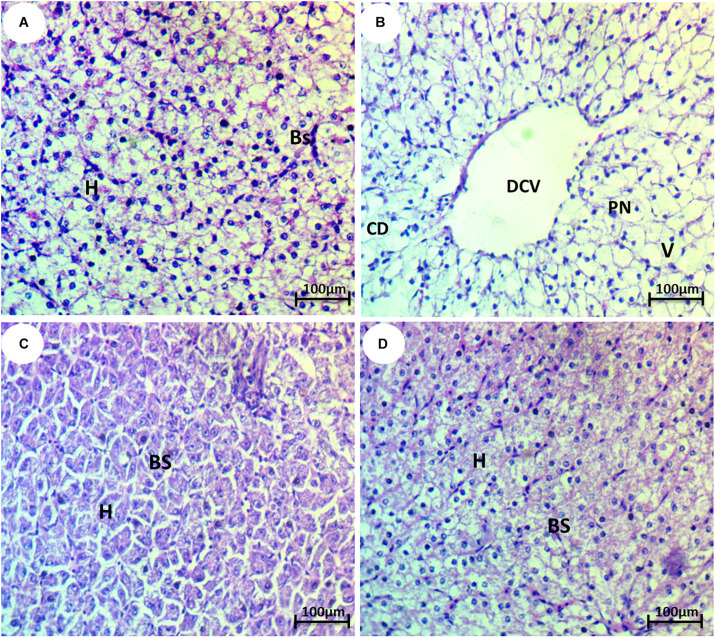
Photomicrograph of liver tissue of the African catfish (*Clarias gariepinus*) showing **(A)** normal architecture of liver hepatocytes (H) and normal blood sinusoids (BS); **(B)** fish exposed to hydroxychloroquine showing complete degeneration (CD), pyknosis of nuclei (PN), dilation of the central vein (DCV), and vacuolization (V); **(C)** fish exposed to hydroxychloroquine + 10 mg/l *Spirulina* showing mild improvement of hepatocytes (H) and blood sinusoids (BS); and **(D)** fish exposed to hydroxychloroquine + 20 mg/l *Spirulina* showing strong improvement of hepatocytes (H) and blood sinusoids (BS) (H&E stain, scale bar 50 μm).

The liver of the control group showed normal hepatocytes and blood sinusoids (Figure 3A). The liver of HCQ-exposed fish showed some histopathological alterations, such as dilation of the central vein, vacuolization, degeneration of hepatocytes, and pyknotic nuclei (Figure 3B). The liver of HCQ-exposed fish and fish co-treated with SP (10 and 20 mg l^–1^ water) showed mild and strong improvement of the histoarchitecture of the liver in a dose-dependent manner (Figures 3C,D).

**FIGURE 4:**
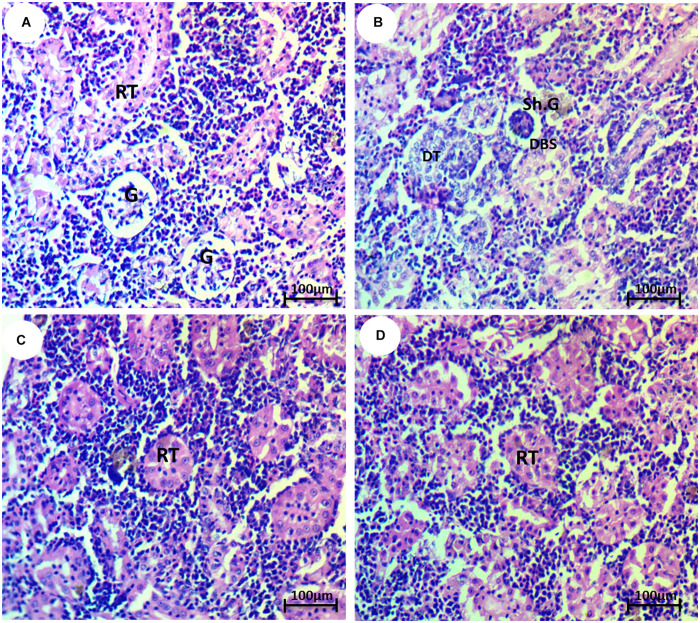
Photomicrograph of kidney tissue of the African catfish (*Clarias gariepinus*) showing **(A)** normal architecture of glomerulus (H) and renal tubule (RT); **(B)** fish exposed to hydroxychloroquine showing degeneration of renal tubule (DT), shrinkage of the glomerulus (Sh.G), and dilation of Bowman’s space (DBS); **(C)** fish exposed to hydroxychloroquine + 10 mg/l *Spirulina* showing mild improvement of the renal tubule (RT); and **(D)** fish exposed to hydroxychloroquine + 20 mg/l *Spirulina* showing moderate improvement of the renal tubule (RT) (H&E stain, scale bar 50 μm).

The normal morphology of glomeruli, renal tubule, and Bowman’s capsule was shown in the kidney tissues of the control group (Figure 4A). The HCQ-exposed group showed several anomalies, such as reduction of glomeruli, dilation of Bowman’s space, and deterioration of renal tubules (Figure 4B). The kidney of HCQ-exposed fish and fish co-treated with SP (10 and 20 mg l^–1^ water) showed mild and moderate improvement of the histoarchitecture of kidney in a dose-dependent manner (Figures 4C,D).

## Discussion

Environmental release and accumulation of pharmaceutical products are a global concern in view of increased awareness of ecotoxicological effects (Soliman et al., 2019; Soliman and Sayed, 2020). In the present study, most of the hematological indices showed a significant decrease after exposure to 3.16 mg/l of HCQ for 15 days in relation to the control group. Besides, Nagaratnam et al. (1978) observed that patients treated with chloroquine for malaria suffered fever and severe anemia and had a palpable spleen, indicating that chloroquine interferes with hemoglobin (Thomé et al., 2013). Current results showed that co-treatment with *Spirulina* significantly inhibits the decrease in the hematological indices induced by HCQ because *S. platensis* consists of iron and vitamins, thus controlling the production of red blood cells (Hemalatha, 2012). *S. platensis* contains polysaccharides which induce RBC regeneration (Mohamed et al., 2014). Moreover, a pigment, phycocyanin, induces the erythropoietin (EPO) hormone which is responsible for erythropoiesis (Zhang, 1994). *Spirulina* contains B-carotene which enhances RBC recovery to decrease cell lysis and alterations (Stivala et al., 1996; Sayed and Soliman, 2018; Hamed et al., 2019b).

The current data showed alterations (increase or decrease) in some biochemical parameters after exposure to 3.16 mg/l of HCQ. The increased level of ALT and AST reflects damage to hepatocytes (Komatsu et al., 2002) or damage in the protein and carbohydrate metabolism (Ramesh et al., 2018). Murugavel and Pari (2004) reported that high doses of chloroquine increased serum uric acid and creatinine which are regarded as biomarkers for renal failure. Co-treatment with *Spirulina* significantly inhibits the increase of these biochemical parameters induced by HCQ, which was sustained by the limited extent of histological changes in the present study. Moreover, Bhat and Madyastha (2001) stated that the existence of blue pigment phycocyanin in *Spirulina* decreases the hepatotoxicity caused by paracetamol-induced free radicals because it contains enzyme SOD, β-carotene, selenium, or vitamins which have immunostimulant activities and protective effects (Alam et al., 2013). The nephroprotective ability of *Spirulina* recorded in the present study may be due to the presence of antioxidant compounds like triterpenes and flavonoids or because of its free radical scavenging effects (Sharoud, 2015).

Antioxidant biomarkers, i.e., TAC, CAT, and SOD, were depleted significantly in the catfish treated with HCQ, which is in accordance with Pari and Murugan (2006), Kumar Mishra et al. (2013), and Asha et al. (2019). A decrease in the activity of CAT may be due to the excess of superoxide anion radical as a consequence of a reduction in the activity of SOD (Kumar Mishra et al., 2013). Besides, the decreased activities of SOD and CAT may be due to decreased synthesis of enzymes or inhibition of enzyme activity by enhanced lipid peroxidation after stress (Murugavel and Pari, 2004). Co-treatment with *Spirulina* increased the levels of antioxidants significantly in HCQ-treated groups. This indicated the ability of *Spirulina* to protect from the oxidative stress caused by HCQ for it has been reported to possess strong antioxidant activity (phycocyanin) and to provoke the free radical scavenging enzyme system (Sabina et al., 2009).

In the current study, HCQ triggered a significant increase in the percentage of poikilocytosis, and a nuclear abnormality of RBCs in relation to the control group, which was reported before in patients treated with chloroquine for malaria, showed iso-poikilocytosis and hypochromia (Nagaratnam et al., 1978). HCQ is assumed to interact with erythrocytes and inhibit dehydrogenase of delta-amino levulinic acid, causing plasma membrane disruption and leading to poikilocytosis in fish (Hamed et al., 2019b). Extensive improvement in the alterations after SP treatment was observed, which indicated the protective role of SP against the alterations in erythrocytes. *Spirulina* is described as an antioxidant and anti-mutagenic due to the presence of alpha-tocopherol and beta-carotene (Araldi et al., 2014).

The histopathological evaluation of the liver showed dilation of the central vein, vacuolization, degeneration of hepatocytes, and pyknosis of nuclei, which are in accordance with Pari and Amali (2005) who stated that the histopathological observations in CQ-treated rats showed feathery degeneration and a microvesicular type of fatty generation with sinusoidal dilation and focal necrosis. This could be due to the formation of highly reactive radicals because of the oxidative threat caused by CQ (Pari and Murugan, 2006). Likewise, El Shishtawy et al. (2013) observed sinusoidal congestion, hepatoportal and hydropic degeneration, cloudy swelling, cellular necrosis (karyolysis, karyorrhexis, and nuclear pyknosis), and inflammatory cellular infiltration in chloroquine and HCQ treated-albino rats. Moreover, Ramesh et al. (2018) noticed severe morphological anomalies, such as vacuolization, nuclear degeneration, cellular edema, necrosis, altered hepatocyte, and increased sinusoidal space, which appeared in CQ-treated *Cyprinus carpio* fingerlings. Histopathological results reveal the incidence of degeneration of liver cells of albino rats upon oral administration of chloroquine (Asha et al., 2019).

The histopathological evaluation of the kidney showed a reduction of glomeruli, dilation of Bowman’s space, and deterioration of renal tubules, which is in accordance with Pari and Murugan (2006), who stated the histopathological observations in chloroquine-treated rat kidneys showing multiple foci of hemorrhage and necrosis with cloudy swelling of tubules, which could be due to the formation of highly reactive radicals as a consequence of oxidative threat caused by CQ (Murugavel and Pari, 2004). Besides, El Shishtawy et al. (2013) mentioned that cloudy swelling, vascular congestion, hydropic degeneration, hemorrhage, inflammation, focal tubular necrosis, and hypercellularity of glomeruli were observed in chloroquine- and HCQ-treated albino rats. In addition, Ramesh et al. (2018) noticed various anomalies, such as tubular cell necrosis, thickening of Bowman’s capsule, reduction of glomeruli, glomerular necrosis, tubular degeneration, hyaline droplet degeneration, congestion in the renal parenchyma, cloudy swelling, renal tubular separation, and reduction of lumens, in the kidney of fish exposed to CQ. Co-treatment with *Spirulina* restored the histological structure to a normal state. *Spirulina* reinforces the stabilization of the plasma membrane, thereby preserving the structural integrity of cells as well as the repair of tissue damage (Stivala et al., 1996).

## Conclusion

The present investigation indicates that HCQ induced hematological, biochemical, and histological changes as well as antioxidant depletion and poikilocytosis in catfish. In addition, *Spirulina* remedies the toxic effects of HCQ due to its bioactive components. Therefore, *Spirulina* can be used in aquaculture for protecting fish from pharmaceutical residue side effects.

## Data Availability Statement

The raw data supporting the conclusions of this article will be made available by the authors, without undue reservation.

## Ethics Statement

The animal study was reviewed and approved by the Assiut University committee.

## Author Contributions

All authors listed have made a substantial, direct and intellectual contribution to the work, and approved it for publication.

## Conflict of Interest

The authors declare that the research was conducted in the absence of any commercial or financial relationships that could be construed as a potential conflict of interest.
