# Supplementary figures and images for: Spirulina platensis Alleviated the Hemotoxicity, Oxidative Damage and Histopathological Alterations of Hydroxychloroquine in Catfish (Clarias gariepinus)
Source: Front Physiol. 2021 Jul 6;12:683669. doi: 10.3389/fphys.2021.683669 (PMC8290523; doi:10.3389/fphys.2021.683669)

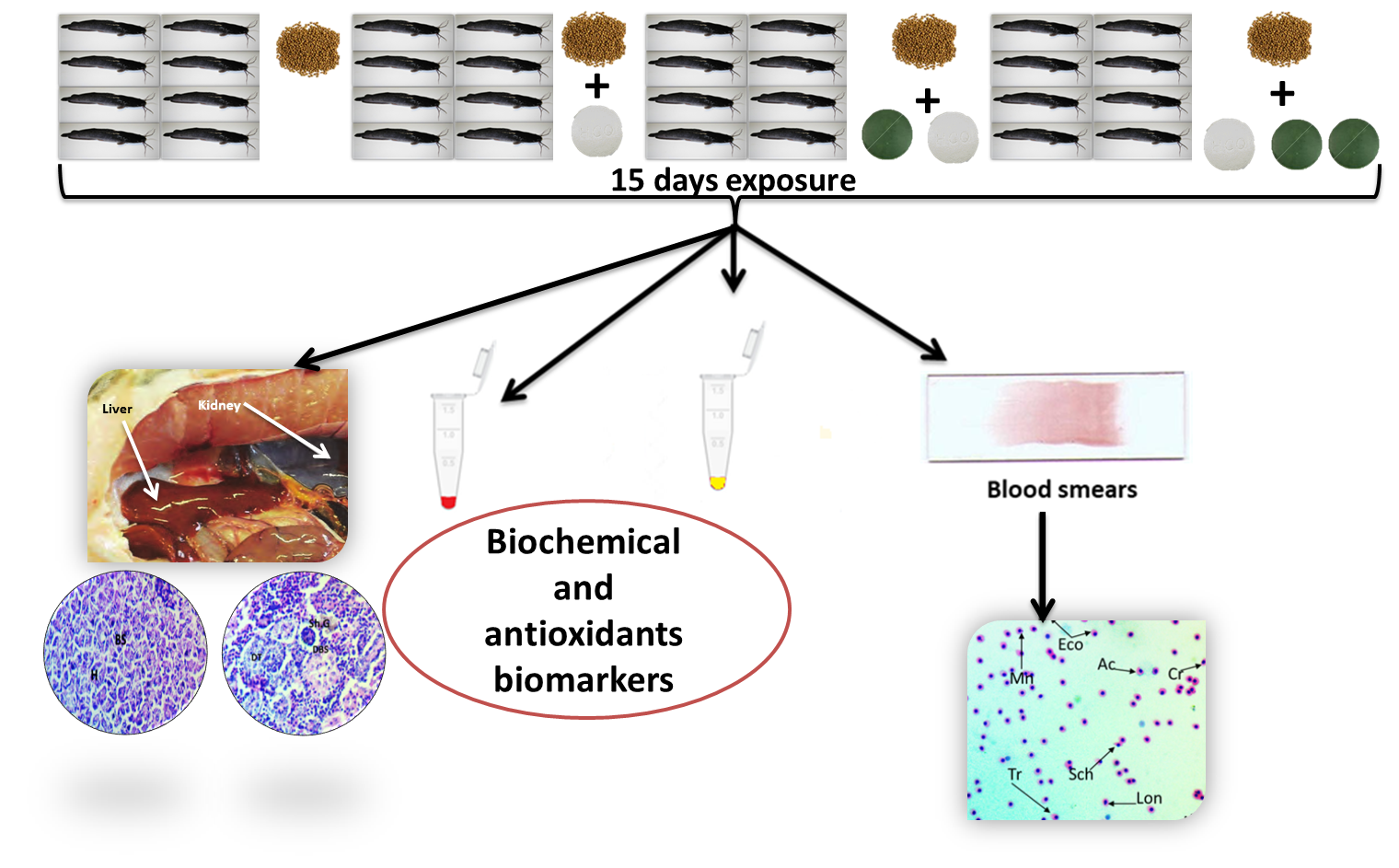

Supplement: Supplementary file 1 [file Image_1.TIF]
